# Effects of endovascular treatment and prognostic factors for recovery of oculomotor nerve palsy caused by posterior communicating artery aneurysms: a multi-center retrospective analysis

**DOI:** 10.1186/s12883-022-02911-y

**Published:** 2022-10-08

**Authors:** Bin Wang, Sheng Liu, Shi-Jie Na, Ya Peng, Wen-Bin Ding, Lin-Bo Zhao, Zhen-Yu Jia, Hai-Bin Shi, Qing Feng

**Affiliations:** 1 Department of Interventional Radiology, The First Affiliated Hospital of Nanjing Medical University, 210029 Nanjing, China; 2 Department of Neurosurgery, Nanjing Drum Tower Hospital, Nanjing University Medical School, 210008 Nanjing, China; 3 Department of Neurosurgery, The Third Affiliated Hospital of Soochow University, 213003 Changzhou, China; 4 Department of Intervention Radiology, the Second Affiliated Hospital of Nantong University, 226001 Nantong, China; 5 Department of Nutrition and Food Hygiene, School of Public Health, Nanjing Medical University, 101 Longmian Avenue, 211166 Nanjing, China

**Keywords:** Oculomotor nerve palsy, Posterior communicating artery aneurysm, Endovascular treatment, Prognostic factor

## Abstract

**Background:**

Oculomotor nerve palsy (ONP) may result from posterior communicating artery (PcomA) aneurysms. We aimed to evaluate the resolution of ONP after endovascular treatment with the intention of clarifying predictors of nerve recovery in a relatively large series.

**Methods:**

A total of 211 patients with ONP caused by PcomA aneurysms underwent endovascular coiling between May 2010 and December 2020 in four tertiary hospitals. We evaluated the demographics, clinical characteristics, aneurysm morphology parameters and ONP resolution to analyze the predictors of ONP recovery using univariate and multivariate analyses.

**Results:**

At the last available clinical follow-up, ONP resolution was complete in 126 (59.7%) patients, partial in 73 (34.6%) patients, and no recovery in 12 (5.7%) patients. The median resolution time after endovascular treatment was 55 days (interquartile range: 40–90 days). In multivariate analysis, degree of ONP (incomplete palsy) on admission (OR 5.396; 95% CI 2.836–10.266; *P* < 0.001), duration of ONP (≤ 14 days) before treatment (OR 5.940; 95% CI 2.724–12.954; *P* < 0.001) were statistically significant predictors of complete recovery of ONP. In the subgroup analysis of patients with unruptured aneurysms, aspirin showed a higher complete recovery rate in univariate analysis (OR 2.652; 95% CI 1.057–6.656; *P* = 0.038).

**Conclusion:**

Initial incomplete ONP and early management might predict better recovery of ONP after endovascular treatment.

## Background

Oculomotor nerve palsy (ONP) is a well-known clinical sign of posterior communicating artery (PcomA) aneurysms, which can be a serious neurologic emergency due to the potential of subarachnoid hemorrhage. ONP occurs in about 20% of patients with PcomA aneurysms [1]. There is no consensus on the optimum therapeutic approach for a PcomA aneurysm with ONP [2–6]. Endovascular therapy has become a popular treatment option for cerebral aneurysms because of its great efficiency and low invasiveness. Approximately half of patients, however, do not recover completely from ONP after endovascular treatment [7, 8].

The probable mechanisms of PcomA aneurysm-related ONP include direct mechanical compression of the third nerve by aneurysm, nerve injury from aneurysm pulsation, and irritation from subarachnoid hemorrhage [3, 7, 9]. Many studies have found that the degree of ONP recovery is influenced by ONP severity, symptom duration, aneurysm morphology, aneurysm status, and treatment modalities [4, 7, 10], however, the sample sizes are mostly small. Furthermore, aneurysm wall inflammation has been found to be related with ONP [11] and there is a case report of complete recovery of optic nerve palsy after anti-inflammatory medication without any treatment for the giant carotid-ophthalmic aneurysm [12]. Aspirin, the most widely used anti-inflammatory, has been shown to beneficially attenuate the aberrant inflammatory microenvironment within the aneurysmal wall [13]. However, as far as we know, there has been no study exploring the effect of aspirin on the recovery of ONP induced by PcomA aneurysm.

In this multi-center retrospective study, we aimed to evaluate the resolution of ONP with the intention of clarifying predictors of nerve recovery in a relatively large series, and to investigate the effect of the aneurysmal morphological parameters and antiplatelet therapy on ONP recovery.

## Methods

### Study design and study population

The research was carried out in accordance with the Declaration of Helsinki (2008) of the World Medical Association, and this study protocol was approved by our institution’s Ethics Committee. Informed consent of the procedure was waived for this retrospective study. The data of patients with aneurysmal ONP who received endovascular treatment for both ruptured and unruptured PcomA aneurysms at four tertiary hospitals was collected from January 2012 to December 2020. Complete ONP was defined as the combination of ptosis, fixed mydriasis, diplopia, and ophthalmoplegia. Incomplete ONP was defined as any combination of these signs, but not all four signs. Patients without follow-up information were eliminated from the study. A total of 211 patients were included in this study. Clinical characteristics associated with ONP recovery were reviewed and analyzed (Table 1).

**Table 1:** Univariate analysis of variables for ONP recovery (n = 211)

Variables	Complete recovery (N = 126)	Unpleasant recovery (N = 85)	OR (95% CI)	p-value
Age (X ± SD)	60.06 ± 11.11	62.05 ± 11.34	0.984 (0.960–1.009)	0.208
Female	105 (83.3%)	71 (83.5%)	0.986 (0.470–2.067)	0.970
Diabetes	7 (5.6%)	8 (9.4%)	0.566 (0.197–1.625)	0.290
Hypertension	70 (55.6%)	49 (57.6%)	0.918 (0.527–1.601)	0.764
Smoking	9 (7.1%)	8 (9.4%)	0.740 (0.274–2.002)	0.554
Alcohol abuse	8 (6.3%)	7 (8.2%)	0.755 (0.263–2.167)	0.602
Subarachnoid hemorrhage	80 (63.5%)	34 (40.0%)	2.609 (1.482–4.592)	0.001
Modified Fisher Scale			1.664 (1.161–2.386)	0.006
0	46 (36.5%)	51 (60.0%)		
1–2	46 (36.5%)	18 (21.2%)		
3–4	34 (27.0%)	16 (18.8%)		
Hunt-Hess Grades			1.973 (1.274–3.057)	0.002
0	46 (36.5%)	51 (60.0%)		
1–2	62 (49.2%)	27 (31.8%)		
3–4	18 (14.3%)	7 (8.2%)		
PDO (≤ 14 days)	112 (88.9%)	54 (63.5%)	4.593 (2.258–9.339)	<0.001
Stent assisted	63 (50.0%)	47 (55.3%)	0.809 (0.466–1.404)	0.450
Raymond scale			1.119 (0.682–1.838)	0.656
1	93 (73.8%)	65 (76.5%)		
2	26 (20.6%)	16 (18.8%)		
3	7 (5.6%)	4 (4.7%)		
Aspirin	73 (57.9%)	49 (57.6%)	1.012 (0.580–1.766)	0.967
Mecobalamine	51 (40.5%)	43 (50.6%)	0.664 (0.382–1.156)	0.148
PDP (incomplete)	93 (73.8%)	33 (38.8%)	4.441 (2.462–8.010)	<0.001
DP (Posterior-lateral-inferior)	52 (41.3%)	20 (23.5%)	0.438 (0.237–0.809)	0.008
Daughter sac	39 (31.0%)	35 (41.2%)	0.640 (0.361–1.137)	0.128
Maximum size (mm)	6.377 ± 2.676	6.690 ± 2.758	0.958 (0.866–1.061)	0.410
AR (Aspect Ratio)	1.395 ± 0.722	1.283 ± 0.700	1.256 (0.842–1.873)	0.264
SR (Size Ratio)	1.809 ± 1.257	1.823 ± 1.225	0.991 (0.794–1.237)	0.934

*PDO, Preoperative duration of ONP; PDP, Preoperative degree of palsy; DP, Dome projectio*n

## Aneurysm direction

According to the method described in Matsukawa et al’s study, the direction of the aneurysm dome around the PComA was classified into 5 directions: superior, posterior, inferior, medial, and lateral [14]. Because oculomotor nerve usually goes parallel to posterior communicating artery along its inferior-lateral side [15], and a PcomA aneurysm projecting inferior-laterally is more likely to compress the oculomotor nerve, the aneurysms were divided into two categories: posterior-lateral-inferior direction and others.

## Treatment and imaging follow-up

All procedures were performed under general anesthesia. Three-dimensional (3D) rotational angiography was used to assess aneurysmal configuration. Each patient received 70 IU/kg of intravenous heparin during procedure, with an additional 1000 IU heparin administered every hour to maintain heparinization. The aneurysm was packed with coils as densely as possible after the microcatheter was advanced into it. For the treatment of wide-necked aneurysms, a stent-assisted coiling technique was used. Double microcatheter technique was used as required.

For unruptured aneurysm, dual antiplatelets were administered daily for 3 to 6 months, then mono-antiplatelet for at least 6 months in patients treated with stent protection. Antiplatelet medications were not routinely prescribed for maintenance in patients treated with coiling alone. It should be noted that aspirin was prescribed for patients with hypertension or atherosclerosis regardless of stenting or not. For rupture aneurysm, if a stent was anticipated after 3D angiography, patients immediately received 300 mg of clopidogrel and 300 mg of aspirin via an orogastric tube or intraoperative intravenous tirofiban (5 µg/kg) and a maintenance dose of 0.1 µg/kg/min for 24 h. Patients were given dual antiplatelet medication (aspirin 100 mg/day for 1 year and clopidogrel 75 mg/day for 3–6 months) after procedure. At the discretion of each doctor, patients were prescribed or not prescribed mecobalamin tablets for at least one month.

The Raymond classification was used to grade the angiographic results. Follow-up angiography was performed at about 6 months after the procedure, and magnetic resonance angiography or angiography annually thereafter.

## Recovery of ONP

ONP was assessed in the clinic. Complete recovery of ONP were defined as: (1) patients did not report diplopia in all directions of gazes; (2) complete resolution of ptosis; (3) full range of movement in medial, downward, and upward gaze; and (4) partial or complete recovery of pupillary reaction. Partial recovery was defined as the resolution of some but not all of the initially present symptoms [10, 16]. The unpleasant recovery group included partial recovery and no recovery. The recovery time of ONP was defined as the period between procedure and ONP recovery (either complete recovery, or partial recovery that was stable with no additional improvement).

### Statistical analysis

Continuous variables were summarized as means ± standard deviation if normally distributed, or median and interquartile ranges if skew distribution. Categorical variables were presented as percentages. Appropriate statistical tests including Fisher’s exact test, Chi-squared tests, or Student’s t-tests were used to determine the factors related to ONP recovery. Factors predictive of ONP recovery in a univariate analysis (*P* < 0.2) were considered potentially independent variables and subsequently included in a multivariate logistic regression analysis. SPSS 23.0 software was utilized for statistical analysis. A *P* < 0.05 was considered statistically significant.

## Results

### Baseline characteristics and procedure outcomes

Of the 211 patients, the mean age was 60.8 ± 11.2 years old (range, 34–95 years). 176 (83.4%) were female. A ruptured aneurysm was found in 114 individuals (54.0%), while an unruptured aneurysm was found in 97 patients (46.0%). All patients received successful endovascular treatment, with 101 patients (47.9%) receiving coiling alone and 110 patients (52.1%) receiving stent-assisted coiling. Raymond class 1 was achieved in 158 patients (74.9%), Raymond class 2 in 42 patients (19.9%), and Raymond class 3 in 11 patients (5.2%).

## Predictors of ONP recovery

At admission, 85 (40.3%) patients had complete ONP and 126 (59.7%) had incomplete ONP. The median interval time between onset of ONP and endovascular procedure was 6 days (interquartile range: 2–12 days). Median follow-up time was 12.7 months (interquartile range: 8.1–18.0 months). At the last available clinical follow-up, ONP resolution was complete in 126 (59.7%) patients, partial in 73 (34.6%) patients, and no recovery in 12 (5.7%) patients. ONP aggravation was not observed immediately after embolization. The median resolution time after endovascular treatment was 55 days (interquartile range: 40–90 days).

In univariate analysis, subarachnoid hemorrhage, preoperative degree of ONP, preoperative duration of ONP, and aneurysm dome projection were all found to be significantly correlated with ONP outcome (Table 1). In a multivariate analysis, preoperative degree of ONP (incomplete palsy) and preoperative duration of ONP (≤ 14 days) were revealed to be independent predictors of complete nerve recovery following procedure (*P*<0.001 respectively) (Table 2).

**Table 2:** Multivariate logistic regression analysis for complete recovery of ONP

Independent factors	OR	95% CI	P-value
Preoperative duration of ONP (≤ 14 days)	5.940	2.724–12.954	<0.001
Preoperative degree of palsy (incomplete)	5.396	2.836–10.266	<0.001

## Effect of aspirin on ONP recovery

Overall, 58.9% (73/122) of patients who took aspirin recovered completely from ONP, and 59.6% (53/89) of patients who did not take aspirin achieved a complete recovery of ONP (*P* = 0.967). In the subgroup analysis of unruptured PcomA aneurysms, the complete recovery rate of ONP was significantly higher in patients taking aspirin than in patients not taking aspirin in univariate analysis (*P* = 0.038), but there was no significant difference in the multivariate analysis (Table 3).

**Table 3:** Univariate and multivariate analysis of variables for ONP recovery in patients with unruptured aneurysm (n = 97)

Variables	Complete recovery (N = 46)	Unpleasant recovery (N = 51)	univariate			multivariate		
			**P value**	**OR (95% CI)**		**P value**	**OR (95% CI)**	
Age (X ± SD)	62.46 ± 11.06	61.06 ± 12.11	0.552	1.011 (0.976–1.046)				
Female	35 (76.1%)	44 (86.3%)	0.202		0.506 (0.178–1.441)			
Diabetes	3 (6.5%)	3 (5.9%)	0.896		1.116 (0.214–5.826)			
Hypertension	27 (58.7%)	28 (54.9%)	0.707		1.167 (0.522–2.616)			
Smoking	4 (8.7%)	4 (7.8%)	0.897		1.119 (0.263–4.757)			
Alcohol abuse	5 (10.9%)	4 (7.8%)	0.609		1.433 (0.361–5.695)			
Preoperative Duration of ONP (≤ 14 days)	36 (78.3%)	26 (51.0%)	0.006		3.462 (1.421–8.430)	0.003	4.463 (1.659–12.009)	
Stent assisted	29 (63.0%)	29 (56.9%)	0.536		1.294 (0.572–2.926)			
Raymond scale			0.648		1.191 (0.563–2.520)			
1	34 (73.9%)	40 (78.4%)						
2	10 (21.7%)	9 (17.6%)						
3	2 (4.3%)	2 (3.9%)						
Aspirin	37 (80.4%)	31 (60.8%)	0.038		2.652 (1.057–6.656)	0.129	2.187 (0.797–6.001)	
Mecobalamine	29 (63.0%)	32 (62.7%)	0.976		1.013 (0.444–2.311)			
Preoperative degree of Palsy (incomplete)	30 (65.2%)	18 (35.3%)	0.004		3.437 (1.491–7.926)	0.004	4.041 (1.579–10.339)	
Dome projection (Posterior-lateral-inferior)	32 (69.6%)	40 (78.4%)	0.321		0.629 (0.251–1.572)			
Daughter sac	15 (57.1%)	20 (42.9%)	0.499		0.750 (0.326–1.727)			
Maximum size (mm)	6.453 ± 2.457	6.618 ± 2.803	0.756		0.976 (0.838–1.137)			
AR (Aspect Ratio)	1.355 ± 0.758	1.386 ± 0.771	0.842		0.948 (0.559–1.606)			
SR (Size Ratio)	1.450 ± 0.964	1.813 ± 1.338	0.139		0.757 (0.524–1.094)	0.284	0.799 (0.530–1.204)	

## Remaining symptoms in unpleasant recovery patients

Among the 211 patients, 183 had ptosis, 148 had fixed mydriasis, 156 had diplopia, and 172 had ophthalmoplegia. At the last available clinical follow-up, partial ONP recovery or no recovery was observed in 85 patients. The remaining symptoms included 13 ptosis, 47 fixed mydriasis, 23 diplopia, and 19 ophthalmoplegia. the symptom of fixed mydriasis displayed worse recovery than other symptoms after treatment (*P* < 0.001).

## Discussion

In this multi-center retrospective study, after endovascular treatment of PcomA aneurysm, the complete recovery rate of ONP was 59.7% and the partial recovery rate was 34.6%, resulting in an overall recovery rate of 94.3%. In our findings, incomplete ONP at admission and early management were independent predictors of complete nerve recovery following endovascular treatment. In the subgroup analysis of patients with unruptured aneurysms, aspirin showed a higher complete recovery rate in univariate analysis.

After the publication of the International Subarachnoid Aneurysm Trial study in 2006, treatment of ruptured aneurysms has swayed toward endovascular treatment. However, there is still debate about the efficiency of surgical clipping and endovascular coiling on the resolution of ONP induced by PcomA aneurysms [2, 4]. Some authors believe that clipping is preferable to clipping because the aneurysmal mass effect, which was thought to be the main pathogenesis of aneurysmal ONP, can be reduced during surgical clipping [2, 17, 18]. The rate of complete ONP resolution has been reported from 32 to 85% after clipping [2]. However, other researches have suggested that pulsatile stimulation of aneurysms may be the main pathogenesis of ONP. Although endovascular therapy can not relieve the mass effect, it was as effective as clipping for the recovery of ONP by reducing the pulsatile stimulation of the aneurysm. They compared the clinical outcome of ONP after coiling and clipping, and found there was no significant difference between two groups, with the rate of complete ONP resolution ranging from 60.3 to 62.5% in coiling group vs. 48.7-87.5% in clipping group [4, 6, 8]. In this study, our results showed the complete recovery rate of ONP was 59.7% and the partial recovery rate was 34.6%, resulting in an overall recovery rate of 94.3% after endovascular treatment, which was in line with other studies. Theoretically, compared to conventional coiling, flow diversion (FD) without coiling or loose coiling can reduce mass effect, which may be more beneficial for the recovery of ONP. However, there are only limited cases using FD for treatment PcomA aneurysms with ONP reported in the literatures and the evidence to support the superiority of FD over conventional coiling is still insufficient [19, 20]. One possible reason for neurointerventionlists hesitating to use FD in ruptured aneurysms is the need of antiplatelet therapy, which is considered dangerous if aneurysms without coiling or loose coiling in acute stage. It is also not a safe choice for unruptured PcomA aneurysms with ONP, considering aneurysms with ONP are usually unstable and have a high risk of rupture.

In our findings, incomplete ONP at admission and early management were independent predictors of complete nerve recovery following endovascular treatment. Despite the fact that they appeared to be simple, our findings were consistent with those of many other studies. Several studies and meta-analyses showed that patients with incomplete ONP had a higher rate of recovery [7, 8, 10, 21]. Others discovered a link between early treatment and the degree of ONP recovery [2, 8, 22, 23]. Mechanical compression was considered as a major factor of aneurysm related ONP, and morphological characteristics of aneurysm might be related with the occurrence and outcome of ONP. According to Lv et al., PcomA aneurysms with ONP showed a distinct morphological-hemodynamic pattern, such as larger size, more irregular shape, and lower wall shear stress [24]. Hall et al. found that patients who presented with an aneurysm < 7 mm had a higher rate of complete palsy resolution compared to aneurysms > 7 mm [7]. However, in other systematic review, aneurysm size was not found to be a significant factor of ONP recovery [18]. The aneurysmal direction might affect the occurrence and recovery of ONP anatomically. Abdurahman et al. reported that the non-posterolateral direction of the aneurysm showed a tendency towards better recovery compared to the posterolateral projection [25], while in another study there was no correlation between aneurysmal direction and ONP recovery [26]. In our study, posterior-lateral-inferior direction of aneurysm dome showed a tendency towards unpleasant ONP recovery compared to other directions in univariate analysis, however, there was no significant difference in multivariate analysis. This result could be caused by anatomical differences between individuals.

Inflammation of the aneurysm wall may be a potential cause of ONP [11]. Animal experiment has verified that aneurysm wall enhancement in magnetic resonance vessel wall imaging is associated with inflammation [27]. Unruptured intracranial aneurysms with ONP or sentinel headache more frequently showed aneurysm wall enhancement than asymptomatic ones [11]. Therefore, anti-inflammatory treatment might contribute to the resolution of cranial nerve palsy. Corticosteroid as an anti-inflammatory medication is widely used for nerve palsy. However, studies focusing on ONP caused by intracranial aneurysms are limited, except for some case reports. Myriam et al. reported a patient with optic nerve palsy caused by a massive carotid-ophthalmic aneurysm [12]. Except for steroids, the patients refused any treatment for aneurysm. After a year, the patient’s optic nerve palsy had completely resolved, and the aneurysm wall enhancement had greatly diminished. Belotti et al. reported a case of ONP caused by neurovascular conflict [28]. In this case, the posterior communicating artery caused a compression of the ipsilateral oculomotor nerve. ONP completely recovered 13 days after the beginning of the steroid treatment. These findings suggested that ONP could be induced by aneurysm wall inflammation or an inflammatory environment around the oculomotor nerves, and that anti-inflammatory medication could contribute to the resolution of cranial nerve palsy.

Aspirin as a kind of antiplatelet drugs has an anti-inflammatory effect and has been confirmed to reduce aneurysm wall inflammation [13]. As we know, there was no study investigating effect of aspirin on ONP up to now. In our study, aspirin was not found to be a predictor of complete nerve recovery either in univariate or multivariate logistic regression analysis. However, in the subgroup analysis, aspirin was revealed to be a statistically significant predictor of complete nerve recovery in patients with unruptured aneurysms in univariate analysis, but not in patients with ruptured aneurysms in subgroup analysis. It might be explained by the probably different pathologic mechanisms of ONP between ruptured and unruptured aneurysms. Apart from mass effect and pulsation irritation, the hemorrhagic irritation might be an inescapable factor in ruptured aneurysms, nevertheless, the inflammation might be a major factor in unruptured aneurysms. According to our preliminary result, aspirin might promote the recovery of ONP for patients with unruptured PcomA aneurysms. This finding maybe advances our understanding of the pathogenesis of aneurysmal ONP, however, further studies are needed to verify the effect of aspirin on ONP recovery.

There are a few limitations in this research. Firstly, both ruptured and unruptured PcomA aneurysms were included in our study. The mechanisms of ONP induced by unruptured PcomA aneurysms were not identical to those generated by ruptured PcomA aneurysms, which may reduce the comparability of the two groups. Secondly, we discovered that the aneurysmal dome’s posterior-lateral-inferior orientation may compromise ONP recovery, but there was no gross pathological or imaging data to confirm whether aneurysms’ posterior-lateral-inferior orientation exacerbated the mass effect in this investigation. Thirdly, we did not perform high-resolution wall imaging to assess the extent of aneurysm wall enhancement during follow-up.

## Conclusion

In this study, we presented the results of a series of 211 patients undergoing endovascular treatment for PcomA aneurysms with ONP. We discovered that more than 90% of patients had varying degrees of ONP recovery after procedures. Preoperative incomplete ONP and early management were the independent factors predicting complete recovery of ONP.

## Data Availability

The datasets used and/or analyzed during the current investigation are available upon reasonable request from the corresponding author.

## Abbreviations

ONP: Oculomotor nerve palsy
PcomA: Posterior communicating artery
3D: Three-dimensional
FD: Flow diversion

## References

[1] Zu QQ, Liu XL, Wang B, Zhou CG, Xia JG, Zhao LB (2017). Recovery of oculomotor nerve palsy after endovascular treatment of ruptured posterior communicating artery aneurysm. Neuroradiology.

[2] Tan H, Huang G, Zhang T, Liu J, Li Z, Wang Z (2015). A retrospective comparison of the influence of surgical clipping and endovascular embolization on recovery of oculomotor nerve palsy in patients with posterior communicating artery aneurysms. Neurosurgery.

[3] Zheng F, Dong Y, Xia P, Mpotsaris A, Stavrinou P, Brinker G (2017). Is clipping better than coiling in the treatment of patients with oculomotor nerve palsies induced by posterior communicating artery aneurysms? A systematic review and meta-analysis. Clin Neurol Neurosurg.

[4] Signorelli F, Pop R, Ganau M, Cebula H, Scibilia A, Gallinaro P (2020). Endovascular versus surgical treatment for improvement of oculomotor nerve palsy caused by unruptured posterior communicating artery aneurysms. J Neurointerv Surg.

[5] Jha V, Sinha V, Abhijit V, Jha N, Singh S. Comparative analysis of the risk factors influencing recovery of function from Oculomotor nerve Palsy in unruptured and ruptured Posterior Communicating Artery Aneurysms. Turk Neurosurg. published online ahead of print, 2021 Oct 19. 10.5137/1019-5149.JTN.32677-20.1 34664686

[6] Sun Z, Yan X, Li X, Wu J (2020). Evaluation of Surgical Clipping and Endovascular Coiling on Oculomotor Nerve Palsy Caused by Internal Carotid Artery Aneurysm. Front Neurol.

[7] Hall S, Sadek AR, Dando A, Grose A, Dimitrov BD, Millar J (2017). The Resolution of Oculomotor Nerve Palsy Caused by Unruptured Posterior Communicating Artery Aneurysms: A Cohort Study and Narrative Review. World Neurosurg.

[8] Zhong W, Zhang J, Shen J, Zhang P, Wang D, Su W (2019). Posterior communicating aneurysm with oculomotor nerve palsy: Predictors of nerve recovery. J Clin Neurosci.

[9] McCracken DJ, Lovasik BP, McCracken CE, Caplan JM, Turan N, Nogueira RG (2015). Resolution of Oculomotor Nerve Palsy Secondary to Posterior Communicating Artery Aneurysms: Comparison of Clipping and Coiling. Neurosurgery.

[10] Chalouhi N, Theofanis T, Jabbour P, Dumont AS, Gonzalez LF, Starke RM (2013). Endovascular treatment of posterior communicating artery aneurysms with oculomotor nerve palsy: clinical outcomes and predictors of nerve recovery. AJNR Am J Neuroradiol.

[11] Fu Q, Wang Y, Zhang Y, Zhang Y, Guo X, Xu H (2021). Qualitative and Quantitative Wall Enhancement on Magnetic Resonance Imaging Is Associated With Symptoms of Unruptured Intracranial Aneurysms. Stroke.

[12] Edjlali M, Boulouis G, Derraz I, Ben Hassen W, Rodriguez-Regent C, Trystram D (2018). Intracranial aneurysm wall enhancement decreases under anti-inflammatory treatment. Neurology.

[13] Roa JA, Zanaty M, Ishii D, Lu Y, Kung DK, Starke RM (2020). Decreased contrast enhancement on high-resolution vessel wall imaging of unruptured intracranial aneurysms in patients taking aspirin. J Neurosurg.

[14] Matsukawa H, Fujii M, Akaike G, Uemura A, Takahashi O, Niimi Y (2014). Morphological and clinical risk factors for posterior communicating artery aneurysm rupture. J Neurosurg.

[15] Zhang WG, Zhang SX, Wu BH (2002). A study on the sectional anatomy of the oculomotor nerve and its related blood vessels with plastination and MRI. Surg Radiol Anat.

[16] Chen PR, Amin-Hanjani S, Albuquerque FC, McDougall C, Zabramski JM, Spetzler RF (2006). Outcome of oculomotor nerve palsy from posterior communicating artery aneurysms: comparison of clipping and coiling. Neurosurgery.

[17] Tian LQ, Fu QX (2020). Recovery of posterior communicating artery aneurysm induced oculomotor nerve palsy: a comparison between surgical clipping and endovascular embolization. BMC Neurol.

[18] Guresir E, Schuss P, Setzer M, Platz J, Seifert V, Vatter H (2011). Posterior communicating artery aneurysm-related oculomotor nerve palsy: influence of surgical and endovascular treatment on recovery: single-center series and systematic review. Neurosurgery.

[19] Binyamin TR, Dahlin BC, Waldau B (2016). Resolution of third nerve palsy despite persistent aneurysmal mass effect after flow diversion embolization of posterior communicating artery aneurysms. J Clin Neurosci.

[20] Szikora I, Marosfoi M, Salomvary B, Berentei Z, Gubucz I (2013). Resolution of mass effect and compression symptoms following endoluminal flow diversion for the treatment of intracranial aneurysms. AJNR Am J Neuroradiol.

[21] Su Z, Shi W, Ge H, Li Y (2019). Efficacy of endovascular intervention in patients with unruptured posterior communicating artery aneurysm-related oculomotor nerve palsy. Neuro Endocrinol Lett.

[22] Leivo S, Hernesniemi J, Luukkonen M, Vapalahti M (1996). Early surgery improves the cure of aneurysm-induced oculomotor palsy. Surg Neurol.

[23] Yang MQ, Wang S, Zhao YL, Zhang D, Zhao JZ (2008). Postoperative recovery from posterior communicating aneurysm complicated by oculomotor palsy. Chin Med J (Engl).

[24] Lv N, Yu Y, Xu J, Karmonik C, Liu J, Huang Q (2016). Hemodynamic and morphological characteristics of unruptured posterior communicating artery aneurysms with oculomotor nerve palsy. J Neurosurg.

[25] Abdurahman E, Amod K, Royston D, Harrichandparsad R (2020). Recovery of oculomotor nerve palsy after endovascular management of posterior communicating artery aneurysms. SA J Radiol.

[26] Patel K, Guilfoyle MR, Bulters DO, Kirollos RW, Antoun NM, Higgins JN (2014). Recovery of oculomotor nerve palsy secondary to posterior communicating artery aneurysms. Br J Neurosurg.

[27] Larsen N, von der Brelie C, Trick D, Riedel CH, Lindner T, Madjidyar J (2018). Vessel Wall Enhancement in Unruptured Intracranial Aneurysms: An Indicator for Higher Risk of Rupture? High-Resolution MR Imaging and Correlated Histologic Findings. AJNR Am J Neuroradiol.

[28] Belotti F, Zanin L, Fontanella MM, Panciani PP (2020). The oculomotor neurovascular conflict: Literature review and proposal of management. Clin Neurol Neurosurg.

